# Readiness of Pediatric General Emergency Department Personnel to Provide Initial Intervention for Suicidal Patient

**DOI:** 10.1192/j.eurpsy.2025.914

**Published:** 2025-08-26

**Authors:** S. Blum, G. Zalsman, S. Yihya

**Affiliations:** 1 Geha Mental Health Center, Petah Tikva, Israel

## Abstract

**Introduction:**

Youth suicide is a significant public health issue, ranking as the third leading cause of death among youth worldwide. Pediatric emergency departments (PEDs) play a critical role as the first line of care for suicidal patients. However, PED personnel, who often lack psychiatric training, need to be equipped with the skills necessary to assess suicidality and provide immediate intervention. This study explores the readiness of non-psychiatric medical personnel in Israeli PEDs to identify and care for suicidal children and adolescents.

**Objectives:**

The primary objective is to evaluate the readiness of non-psychiatric PED personnel to detect and intervene with suicidal youth. Specific objectives include assessing:

1. Frequency of suicidal patient encounters in PEDs.

2. Training and confidence in managing suicidal cases.

3. Experience and self-assessed ability to provide immediate care to suicidal patients.

**Methods:**

This cross-sectional study surveyed 87 non-psychiatric PED medical staff across Israel using a questionnaire distributed through REDCap. The survey included both quantitative questions on a 5-point Likert scale and open-ended qualitative questions. Data were analyzed using descriptive statistics for quantitative responses and thematic analysis for qualitative data.

**Results:**

A majority (76%) reported treating suicidal patients within the past year, though only 35% felt confident in providing care. Training levels were insufficient, with participants scoring an average of 2.25/5 on whether they had received adequate training. Most respondents (45%) expressed a need for further education in pediatric psychiatry, and 60% disagreed with the adequacy of current tools for identifying and managing suicidality. The qualitative analysis revealed a perceived gap in training and preparedness, emphasizing the importance of specialized education.

**Conclusions:**

Israeli PED personnel frequently encounter suicidal youth but often feel underprepared to provide appropriate care. There is a clear need for enhanced training programs, particularly in suicide prevention, to equip medical staff with the tools necessary to effectively identify and treat at-risk patients. Recommendations include curricular updates and specialized workshops for PED personnel to improve suicide intervention outcomes.

**Disclosure of Interest:**

None Declared

